# Which critically ill patients are more susceptible to the adverse effects associated with feeding intolerance? A secondary analysis of a cluster-randomized controlled trial

**DOI:** 10.3389/fnut.2026.1729192

**Published:** 2026-03-23

**Authors:** Youquan Wang, Annika Reintam Blaser, Yanhua Li, Charles Chin Han Lew, Hongxiang Li, Xinyu Li, Lu Ke, Dong Zhang

**Affiliations:** 1 Department of Critical Care Medicine, The First Hospital of Jilin University, Changchun, China; 2 Department of Anaesthesiology and Intensive Care, University of Tartu, Tartu, Estonia; 3 Department of Intensive Care, Lucerne Cantonal Hospital, Lucerne, Switzerland; 4 Department of Dietetics and Nutrition, Ng Teng Fong General Hospital, Singapore, Singapore; 5 Department of Critical Care Medicine, Jinling Hospital, Medical School of Nanjing University, Nanjing, China; 6 National Institute of Healthcare Data Science, Nanjing University, Nanjing, China

**Keywords:** clinical outcomes, feeding intolerance, intensive care unit, mortality, nutritional support

## Abstract

**Objective:**

It remains unclear which critically ill patients are more susceptible to the adverse effects associated with feeding intolerance (FI), which is common in patients receiving early enteral nutrition (EEN). Therefore, we aimed to explore the association between FI and short-term outcomes in critically ill patients receiving EEN.

**Methods:**

This secondary analysis of a multicenter cluster-randomized controlled trial included patients who initiated enteral nutrition within 48 h of admission and received it for at least 3 days. We utilized univariate and multivariate propensity score-weighted logistic regression analyses to investigate the association between FI and 28-day mortality. We conducted multiple subgroup analyses to identify subcohorts that are more likely to be adversely impacted by FI.

**Results:**

A total of 1,545 patients were included in the final analysis, among whom 856 experienced FI and 689 did not. In univariate analyses, no association was found between FI and 28-day mortality [odds ratio (OR) 1.302, 95% CI 0.967–1.752, *p* = 0.082]. After multiple adjustment, we found the occurrence of FI was associated with higher 28-day mortality [adjusted OR (aOR) 1.370, 95% CI 1.002–1.873, *p* = 0.048]. This finding was consistent in the propensity-weighted model (aOR 1.370, 95% CI 1.010–1.873, *p* = 0.015). In multivariate analyses, larger effect estimates were observed among patients aged over 65 years (aOR 1.544, 95% CI 1.019–2.338, *p* = 0.04), with a normal body mass index (BMI) (aOR 1.523, 95% CI 1.065–2.177, *p* = 0.021), with a primary diagnosis of respiratory disease (aOR 1.822, 95% CI 1.149–2.891, *p* = 0.011), and with a sequential organ failure assessment (SOFA) score of ≤ 8 (aOR 1.864, 95% CI 1.204–2.885, *p* = 0.005).

**Conclusion:**

In critically ill patients receiving EEN, FI was associated with higher 28-day mortality, longer ICU LOS, fewer ventilator-free days and ICU-free days. Elderly patients, those with a normal BMI, those with a primary diagnosis of respiratory diseases, and those with a SOFA score of ≤ 8 may be more susceptible to adverse effects of FI. However, these subgroup findings should be interpreted cautiously given multiple comparisons.

## 1 Introduction

For patients who are unable to eat orally, guidelines recommend early enteral nutrition (EN) rather than parenteral nutrition (PN) or delayed nutritional therapy (1, 2). However, in critically ill patients, gastrointestinal (GI) dysfunction may occur to varying degrees, which may hinder the effective delivery of EN (3). When the intensity of EN does not align with the GI function, feeding intolerance (FI) may arise, potentially leading to a reduction or even cessation of EN. On the other hand, FI may also be associated with poorer clinical outcomes (4).

The ESICM guidelines were the first to propose a definition of FI, which involves a comprehensive assessment based on GI symptoms and the feeding dose initiated within 72 h of EN administration (3). Some studies indicate that approximately half of critically ill patients experience FI during the delivery of EN, and FI is associated with poorer clinical outcomes (5–8). Individualized prediction of the likelihood of FI in critically ill patients, followed by the development of tailored nutritional strategies, may reduce the incidence of FI (9). This approach could facilitate the successful delivery of EN, improve nutritional status, promote recovery from illness, and potentially enhance prognosis (10). However, current predictive tools require higher-quality datasets for external validation.

Balancing EN with FI presents a significant challenge for intensive care clinicians. Given the current research and clinical landscape, it appears that FI may not be entirely avoidable in many cases. Moreover, it is not clear to what extent, in which patients and via which mechanism FI may influence outcome. Therefore, we have undertaken an analysis of data from a prospective, multicenter, cluster-randomized study to address the following two questions: 1. Is there an association between FI and poorer short-term clinical outcomes in critically ill patients? This question serves to validate findings from previous studies. 2. Which critically ill patients are more susceptible to adverse effects associated with FI? This question is innovative and exploratory, and it is also the primary focus of this study.

## 2 Methods

### 2.1 Study design and patients

This study presents a secondary analysis of the multicenter, cluster-randomized controlled trial known as the NEED trial (11). In summary, 97 intensive care units (ICUs) across China were randomly assigned to either a guideline group that implemented a feeding protocol or a control group that followed local clinical practices. The feeding guidelines outline the appropriate timing for initiating EN and PN, criteria for assessing FI, and methods for adjusting the feeding rate based on intolerance evaluations to meet the nutritional goals of 25–30 kcal/kg/day for calorie and 1.2–2.0 g/kg/day for protein. A total of 2,772 patients were enrolled within 24 h of ICU admission from 90 ICUs between March 26, 2018, and July 4, 2019. The original study protocol received approval from the ethics committee of Jinling Hospital (22017NZKY-019-02). Written informed consent was obtained from all participants or their next of kin prior to inclusion in the study. Additionally, the trial was registered with the ISRCTN registry (ISRCTN12233792) before the enrollment process began.

Our study included an analysis of a subset of participants based on specific inclusion criteria: (1) initiation of EN within 48 h of ICU admission; and (2) patients with at least 3 days of enteral feeding. We excluded patients classified as acute gastrointestinal injury (AGI) grade IV, as they have a significantly high probability of developing FI and are unable to start EN early. Additionally, we excluded patients who were receiving oral intake, as uncertainty about energy intake could lead to undetermined FI.

### 2.2 Data collection

All data were obtained from electronic databases, including patients’ general clinical information (such as age, sex, body mass index, and number of comorbidities), primary diagnosis, Acute Physiology and Chronic Health Evaluation II (APACHE II) score, Sequential Organ Failure Assessment (SOFA) score, AGI grade, modified nutrition risk in critically ill (mNUTRIC) score, specific details of nutritional delivery (such as the timing of EN initiation, formula used, actual daily feeding volume, and feeding route), as well as GI symptoms and adverse events. For non-obese patients, the actual weight recorded on the day of admission was used to calculate the average daily energy and protein intake. For obese patients (BMI ≥ 30 kg/m^2^), the ideal body weight was used, calculated using the Broca index: height (cm) - 100 (for males) or height (cm) - 105 (for females) (12).

### 2.3 Assessment of FI

We used detailed data to assess the occurrence of FI in critically ill patients during the acute phase of critical illness (within 7 days). FI was diagnosed according to the criteria outlined in the 2012 ESICM guidelines (3). Specifically, FI was defined as (1) GI symptoms [vomiting, a high gastric residual volume (single residual volume greater than 200 mL) or diarrhea (more than 3 loose or liquid stools per day, stool weight exceeding 200–250 g/day or 250 mL/day)] and discontinuation or reduction of EN; and/or (2) 20 kcal/kg/d not reached after 72 h of EN.

YQ Wang and YH Li evaluated the time period of 72 h after EN start and defined EN being present or absent: (1) any GI symptoms; (2) 20 kcal/kg/d not reached after 72 h of EN; (3) both. They evaluated FI separately, and finally integrated the FI results. When the FI evaluation results were inconsistent, HX Li and D Zhang discuss and determine the final FI evaluation results.

### 2.4 Missing data

In this secondary analysis of the NEED trial, we used the dataset as provided by the original study team, including baseline covariates imputed according to the prespecified NEED statistical analysis plan (11).

### 2.5 Statistical analyses

The normality of continuous variables was assessed using the Shapiro–Wilk test. For variables with skewed distributions, the Wilcoxon-Mann–Whitney U-test was used, and results were reported as the median and interquartile range. Categorical variables were described by frequency (percentage) and compared using either Pearson’s chi-squared test or Fisher’s exact test, depending on which was appropriate.

Subsequently, we investigated factors related to FI through both univariate and multivariable analyses. For multivariable model selection, we employed a bidirectional stepwise approach aimed at minimizing the Akaike information criterion (AIC). We evaluated multicollinearity among variables by calculating the variance inflation factor and conducting the Farrar–Glauber test. The goodness of fit was assessed using the Hosmer–Lemeshow test.

Then, we explored the association between FI and 28-day mortality with univariable logistic regression and multivariable regression analysis. To address potential indication bias related to FI, we also conducted multivariable analyses utilizing a propensity score. The likelihood of being in the FI group was modeled using the nonparametric gradient boosting machine learning algorithm from the Twang package (13). Confounding factors included in the propensity score were age, sex, BMI range, and SOFA score. We evaluated the balance of the propensity model by analyzing the standardized effect sizes of the variables. Standardized effects below 0.20 indicated low balance, while 0.40 was considered moderate and 0.60 as large (Supplementary Figure S1). The weights derived from the propensity score were applied in the multivariable logistic regression. This allowed us to assess the association with 28-day mortality in a pseudo-population where the characteristics of subjects with FI and non-FI were balanced. We opted for a double robust approach to minimize bias related to differences in the distribution of confounding factors, which may remain despite propensity score weighting (14). Consequently, we adjusted our propensity-weighted (PW) regression model for all covariates included in the propensity score. Additionally, we explored the connection between FI and other short-term clinical outcomes.

Finally, to evaluate the strength of the association between FI and 28-day mortality in specific subgroups, we conducted a subgroup analysis based on the following variables: age ≤ 65 years or > 65 years, sex (male or female), BMI categories (underweight, normal weight, overweight, obese), primary diagnosis (respiratory, circulatory, others), medical or surgical patients, NEED or control group, admission SOFA score ≤ 8 or > 8, and mNUTRIC score < 5 or ≥ 5. We performed multivariable multilevel logistic regression analyses for each subgroup, the results are presented in the form of a forest plot. Given multiple subgroup comparisons, we adjusted the *p* values from the multivariable-adjusted subgroup analyses using the Benjamini–Hochberg false discovery rate (BH-FDR) procedure. FDR-adjusted *q* values were reported in the Supplementary Table S2, and subgroup findings were interpreted as exploratory. Furthermore, we also considered the impact of the time to the occurrence of the primary endpoint on the statistical analysis. Therefore, we employed Kaplan–Meier survival curves and the Log-rank test to document the survival time up to 28 days after ICU admission.

Statistical analysis was conducted in JMP (SAS Institute) and R version 4.3.1 (R Foundation for Statistical Computing, Vienna, Austria) with RStudio version 1.0.136 (RStudio Inc., Boston, MA, USA).

## 3 Results

### 3.1 Patients baseline characteristics

**Figure 1:**
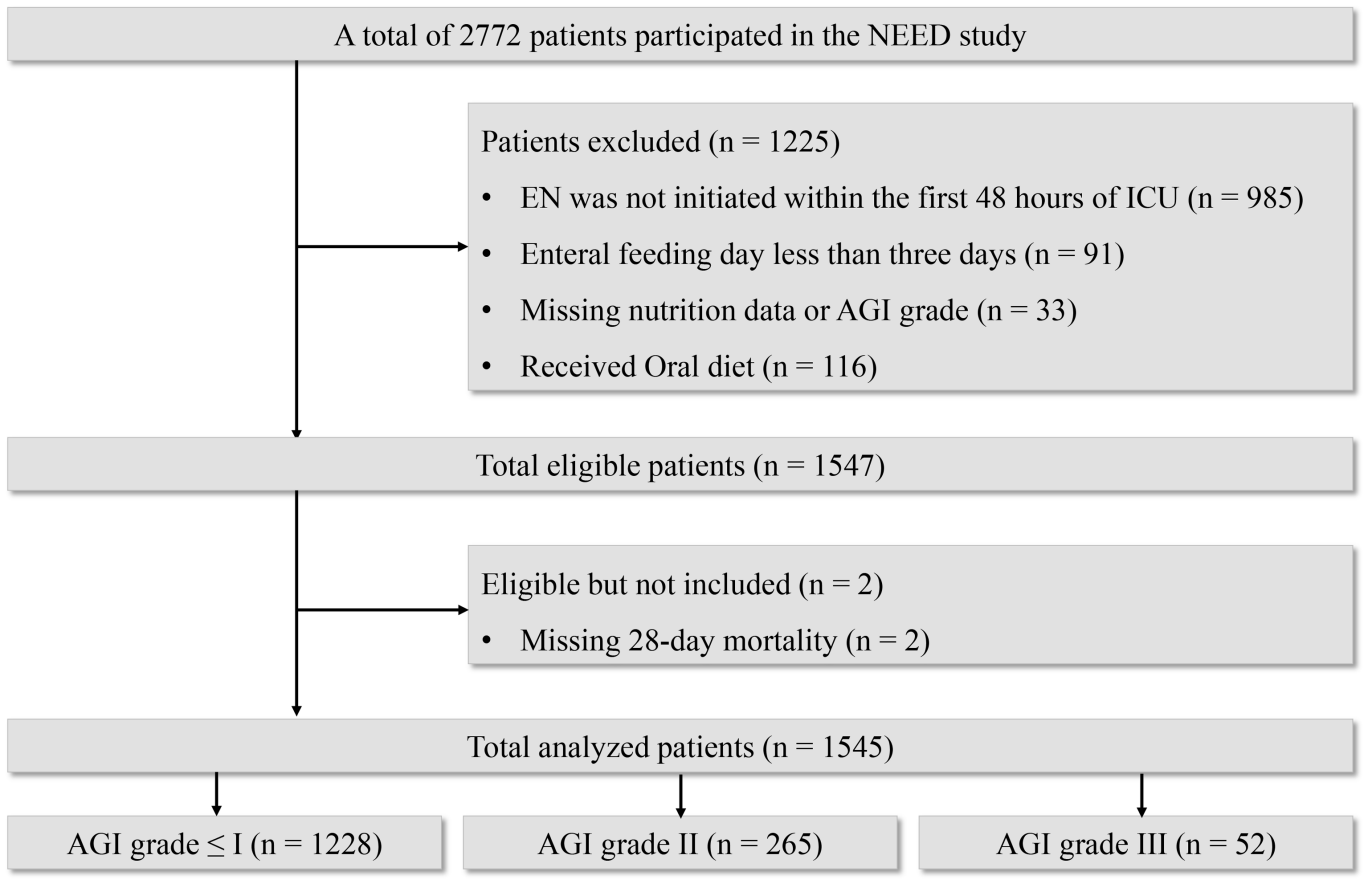
Flowchart of patient selection.

Out of a total of 2,772 patients, 1,545 were eventually included in the analysis (Figure 1). The mean age of the patients was 62.2 ± 17.6 years, and their mean BMI was 22.7 ± 3.2. Among them, 67.1% were male. The median APACHE II score was 18.0 (range: 14.0–22.0), the median SOFA score was 7.0 (range: 5.0–9.0), and the median mNUTRIC score was 4.0 (range: 3.0–6.0). Most patients had mild gastrointestinal injury: 79.5% had AGI grade ≤I, 17.2% had AGI grade II, and 3.4% had AGI grade III. The baseline characteristics of the research objects are summarized in Table 1.

**Table 1:** Baseline characteristics of patients.

Characteristics	Total (*n* = 1,545)	FI (*n* = 856)	Non-FI (*n* = 689)	*p* value
Age, mean (SD), y	62.2 (17.6)	62.4 (17.5)	62.0 (17.8)	0.656
Male, No. (%)	1,036 (67.1)	610 (71.3)	426 (61.8)	<0.001
BMI, mean (SD), kg/m^2^	22.7 (3.2)	23.2 (3.2)	22.1 (3.2)	<0.001
BMI range (%)				<0.001
<18.5	91 (5.9)	23 (2.7)	68 (9.9)	
18.5–25	1,160 (75.1)	647 (75.6)	513 (74.5)	
25–30	225 (16.5)	157 (18.3)	98 (14.2)	
>30	39 (2.5)	29 (3.4)	10 (1.5)	
Primary diagnosis, No. (%)				0.045
Neurologic	266 (17.2)	147 (17.2)	119 (17.3)	
Cardiovascular	374 (24.2)	226 (26.4)	148 (21.5)	
Respiratory	729 (47.2)	377 (44.0)	352 (51.1)	
Multi trauma	32 (2.1)	18 (2.1)	14 (2.0)	
Others	144 (9.3)	88 (10.3)	56 (8.1)	
Critical care related score^a^				
APACHE II, median (IQR)	18.0 (14.0–22.0)	18.0 (15.0–23.0)	18.0 (13.0–22.0)	<0.001
SOFA, median (IQR)	7.0 (5.0–9.0)	7.0 (5.0–10.0)	7.0 (5.0–9.0)	0.005
AGI grade, No. (%)				<0.001
≤I	1,228 (79.5)	608 (71.0)	620 (90.0)	
II	265 (17.2)	202 (23.6)	63 (9.1)	
III	52 (3.4)	46 (5.4)	6 (0.9)	
mNUTRIC, median (IQR)	4.0 (3.0–6.0)	4.0 (3.0–6.0)	4.0 (3.0–5.0)	0.011
Number of co-morbidities, median [IQR]	2 (1–3)	2 (1–3)	2 (1–3)	0.472
Organ support therapy, *n* (%)				
MV^b^, No. (%)	939 (60.8)	536 (62.6)	403 (58.5)	0.099
CRRT^b^, No. (%)	184 (11.9)	115 (13.4)	69 (10.0)	0.039
Vasopressors^a^, No. (%)	396 (25.6)	242 (28.3)	154 (22.4)	0.008
Source of ICU admission, *n* (%)				0.741
Surgical	392 (25.4)	220 (25.7)	172 (25.0)	
Medical	1,153 (74.6)	636 (74.3)	517 (75.0)	
EN delivery within 7 days after enrollment, median [IQR]				
Mean daily EN energy intake, kcal /d	964.3 (714.3–1242.9)	785.7 (535.7–959.3)	1232.1 (1057.1–1428.6)	<0.001
Mean daily EN protein intake, g/d	38.6 (28.6–49.7)	31.4 (21.4–38.4)	49.3 (42.3–57.1)	<0.001
Study interventions, *n* (%)				0.389
NEED group	867 (56.1)	472 (55.1)	395 (57.3)	
Control group	678 (43.9)	384 (44.9)	294 (42.7)	
Tube feeding route, No. (%)				0.986
Prepyloric	1,507 (97.5)	835 (97.5)	672 (97.5)	
Postpyloric	38 (2.5)	21 (2.5)	17 (2.5)	
Results of the first 24 h of testing, median [IQR]				
Albumin, g/L	31.6 (27.8–36.1)	31.2 (27.5–36.0)	32.0 (28.0–36.2)	0.164
Pre-albumin, g/L	0.15 (0.11–0.22)	0.15 (0.11–0.21)	0.16 (0.11–0.23)	0.314
CRP, mg/L	54.5 (16.9–114.0)	57.0 (16.0–119.0)	50.0 (18.0–108.4)	0.252
Lac, mmol/L	1.8 (1.2–2.7)	1.8 (1.2–2.8)	1.7 (1.1–2.7)	0.403

BMI, Body mass index; APACHE II, Acute physiology and chronic health evaluation II; SOFA, Sequential organ failure assessment; AGI, Acute gastrointestinal injury; EN, Enteral nutrition; MV, Mechanical ventilation; CRRT, Continuous renal replacement therapy; FI, Feeding intolerance; IQR, interquartile range. Data presented as mean (standard deviation), median (IQR) or *n* (%).

a Indicated within 24 h of ICU admission.

b Within 7 days of ICU admission.

### 3.2 Results of FI assessment

We conducted an objective assessment of whether each critically ill patient experienced FI (feeding intolerance) based on detailed feeding data. Among the total of 856 patients who developed FI, 38 patients were diagnosed based solely on GI symptoms, 79 patients could not reach at least 20 kcal/kg BW/day via enteral route within 72 h of feeding attempt, and 739 patients met the diagnostic criteria for FI based on both GI symptoms and enteral feeding doses.

**Figure 2:**
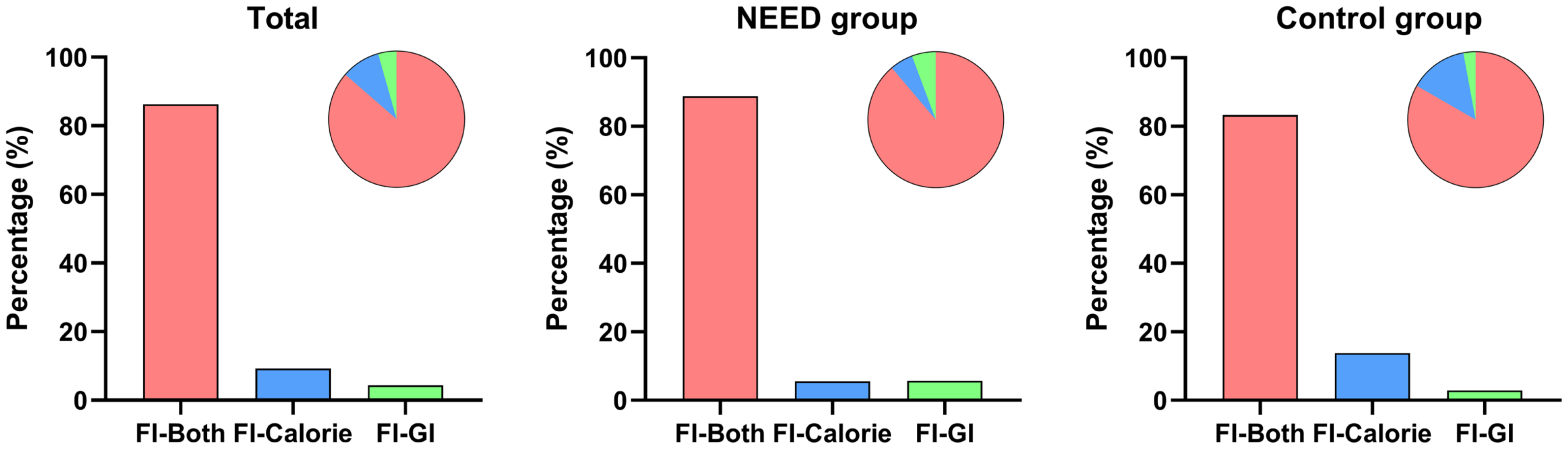
The diagnostic process and proportion of FI in the total data, the NEED group, and the control group. FI-Both: Both GI symptoms and enteral feeding doses met the diagnostic criteria for FI; FI-Calorie: At least 20 kcal/kg BW/day via enteral route cannot be reached within 72 h of feeding attempt, but GI symptoms were not sufficient to diagnose FI; FI-GI: FI was diagnosed based solely on GI symptoms.

We also considered the potential impact of the intervention measures in the cluster-randomized controlled trial on the occurrence of FI. The diagnostic process and proportions of FI in the overall cohort, the NEED group and the control group are shown in Figure 2.

Among the 867 patients in the NEED group, 472 developed FI (incidence rate: 54.4%), while in the control group of 678 patients, 384 developed FI (incidence rate: 56.6%) (Table 1). In the NEED group, the proportion of patients diagnosed with FI based solely on GI symptoms was higher than that in the control group (*p* = 0.044). The proportion of patients diagnosed with FI solely due to insufficient calorie intake was lower in the NEED group than in the control group (*p* < 0.001). The proportion of patients whose diagnosis of FI was based on both GI symptoms and enteral feeding doses was higher in the NEED group than in the control group (*p* = 0.021) (Table 2).

**Table 2:** Nutritional data of patients with feeding intolerance.

Nutritional data	Total (*n* = 856)	NEED group (*n* = 472)	Control group (*n* = 384)	*p* value
EN delivery within 7 days after enrollment, median [IQR]				
Mean daily EN energy intake, kcal /d	785.7 (535.7–959.3)	782.1 (515.0–928.6)	785.7 (564.6–996.4)	0.199
Mean daily EN protein intake, g/d	31.4 (21.4–38.4)	31.3 (20.6–37.1)	31.4 (22.6–39.9)	0.199
AGI grade, No. (%)				0.333
≤I	608 (71.0)	345 (73.1)	263 (68.5)	
II	202 (23.6)	103 (21.8)	99 (25.8)	
III	46 (5.4)	24 (5.1)	22 (5.7)	
Diagnostic basis of FI				
FI-GI^a^, No. (%)	38 (4.4)	27 (5.7)	11 (2.9)	0.044
Gastric retention, No. (%)	20 (2.3)	17 (3.6)	3 (0.8)	0.074
Diarrhea, No. (%)	14 (1.6)	8 (1.7)	6 (1.6)	0.266
Others^b^, No. (%)	3 (0.3)	2 (0.4)	1 (0.3)	1.0
Two or more GI symptoms, No. (%)	1 (0.1)	0 (0)	1 (0.3)	0.289
FI-calorie^c^, No. (%)	79 (9.2)	26 (5.5)	53 (13.8)	< 0.001
FI-both^d^, No. (%)	739 (86.3)	419 (88.8)	320 (83.3)	0.021
Gastric retention, No. (%)	349 (40.8)	197 (41.7)	152 (39.6)	0.896
Diarrhea, No. (%)	222 (25.9)	119 (25.2)	103 (26.8)	0.266
Others^b^, No. (%)	36 (4.2)	21 (4.4)	15 (3.9)	0.839
Two or more GI symptoms, No. (%)	132 (15.4)	82 (17.4)	50 (13.0)	0.165

FI, Feeding intolerance; GI, Gastrointestinal. Data presented as median (IQR) or *n* (%).

a FI was diagnosed based solely on GI symptoms.

b Other GI adverse events.

c At least 20 kcal/kg BW/day via enteral route cannot be reached within 72 h of feeding attempt, but GI symptoms were not sufficient to diagnose FI.

d Both GI symptoms and enteral feeding doses met the diagnostic criteria for FI.

### 3.3 Patients EN intake characteristics

**Figure 3:**
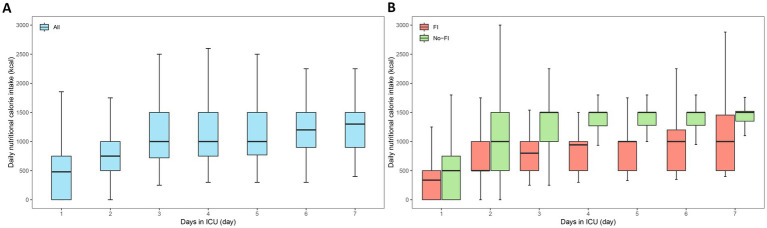
Daily nutritional calorie intake for all patients **(A)**, feeding intolerance (FI) patients and non-FI patients **(B)** during the 1–7 days after intensive care unit (ICU) admission. Intake of calorie was calculated for all days during ICU admission where nutritional intake could be fully quantified. Horizontal lines in boxes represent medians; bottoms of boxes show 25th percentile and tops of boxes show 75th percentile. Ends of whiskers represent the upper adjacent value (ie, 75th percentile plus 1.5 times the IQR) and the lower adjacent value (ie, 25th percentile minus 1.5 times the IQR).

**Figure 4:**
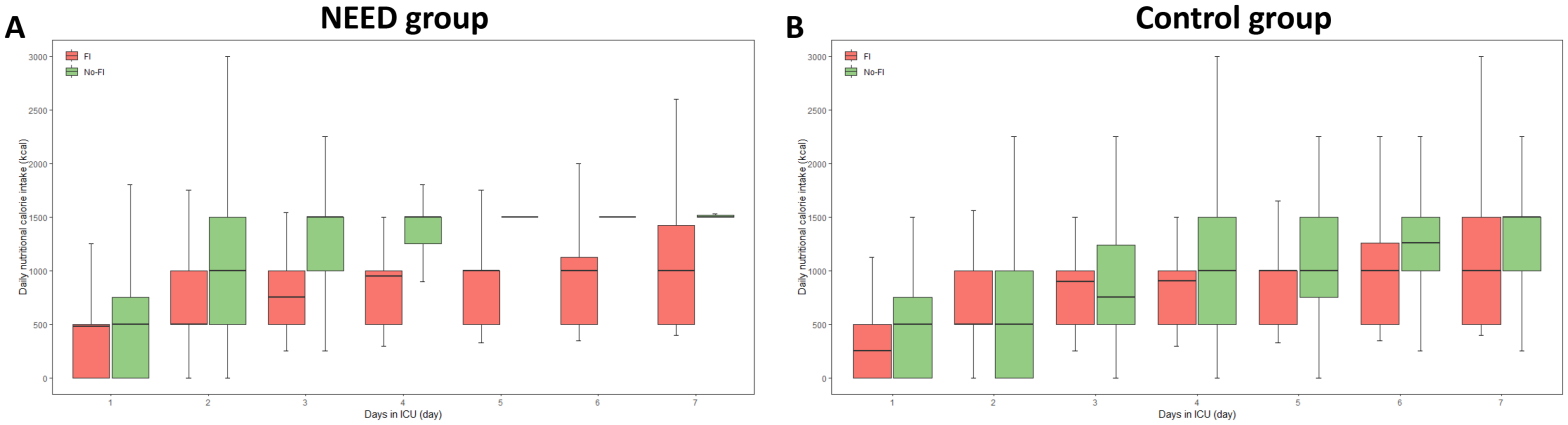
Daily nutritional calorie intake for feeding intolerance (FI) patients and non-FI patients in the NEED group **(A)** and the control group **(B)** during the 1–7 days after intensive care unit (ICU) admission. Intake of calorie was calculated for all days during ICU admission where nutritional intake could be fully quantified. Horizontal lines in boxes represent medians; bottoms of boxes show 25th percentile and tops of boxes show 75th percentile. Ends of whiskers represent the upper adjacent value (i.e., 75th percentile plus 1.5 times the IQR) and the lower adjacent value (i.e., 25th percentile minus 1.5 times the IQR).

The mean daily EN energy intake for all patients during the 7-day ICU stay was 964.3 (714.3–1242.9) kcal/day; the mean daily EN energy intake of patients with FI was 785.7 (535.7–959.3) kcal/day during 7 days in ICU and the mean daily EN energy intake of patients without FI was 1232.1 (1057.1–1428.6) kcal/day during the 7 days in ICU. Compared with non-FI patients, mean daily EN energy intake was significantly lower in patients with FI (*p* < 0.001). Daily nutritional calorie intake for all patients, FI patients and non-FI patients during the 1–7 days after ICU admission were shown in Figure 3. Daily nutritional calorie intake for FI patients and non-FI patients during the 1–7 days after ICU admission in the NEED and control subgroups is shown in Figure 4. Similar to energy intake, daily EN protein intake was significantly lower in FI patients than in non-FI patients [31.4 (21.4–38.4) vs. 49.3 (42.3–57.1), *p* < 0.001].

### 3.4 Factors related to FI occurrence

The results of multi-factor logistic regression showed: Patients who were obese (aOR 10.251, 95% CI 4.377–25.707), overweight (aOR 4.765, 95% CI 2.796–8.359), or of normal weight (aOR 3.717, 95% CI 2.297–6.222), the incidence of FI was higher than that of underweight patients. Compared with male patients, the incidence of FI was lower in female patients. The occurrence of FI was positively associated with SOFA score (Supplementary Table S1).

### 3.5 Association between FI and 28-day mortality

In univariate analyses, no association was found between FI and 28-day mortality (OR 1.302, 95% CI 0.967–1.752, *p* = 0.082). However, in multivariate analyses (aOR 1.370, 95% CI 1.002–1.873, *p* = 0.048), the occurrence of FI was associated with an increased 28-day mortality. This association was observed in the propensity-weighted model (aOR 1.370, 95% CI 1.010–1.873, *p* = 0.015) (Table 3).

**Table 3:** Short-term clinical outcomes of patients.

Outcomes	FI (*n* = 856)	Non-FI (*n* = 689)	OR (95% CI)	*p* value
Primary outcome				
28-day mortality (Crude^a^), No. (%)	128 (15.0)	82 (11.9)	1.302 (0.967–1.752)	0.082
28-day mortality (Multilevel analysis^b^), No. (%)	128 (15.0)	82 (11.9)	1.380 (1.012–1.881)	0.042
28-day mortality (Propensity-weighted cohort^c^), No. (%)	128 (15.0)	82 (11.9)	1.370 (1.010–1.873)	0.015
Secondary outcome				
ICU LOS (days)	16 (9, 28)	14 (9, 28)	1.903 (0.436–3.370)	0.011
ICU-free days D28 (days)	12 (0, 19)	14 (0, 19)	-0.919 (−1.763 – −0.076)	0.033
Ventilator-free days D28 (days)	19.5 (7, 28)	20 (11, 28)	-1.149 (−2.213 – −0.084)	0.035
Albumin D10 (g/L)	32.7 ± 6.7	32.6 ± 6.2	0.119 (−0.654–0.893)	0.762
Pre-albumin D10 (mg/dL)	0.17 (0.12, 0.21)	0.17 (0.12, 0.22)	0.007 (−0.007–0.022)	0.320

FI, Feeding intolerance; ICU, Intensive care unit; LOS, length of stay.

a Univariable logistic regression.

b Multilevel, multivariable analysis with a random effect on the centre of inclusion and adjustment of age, sex, BMI range, and SOFA score.

c Propensity-weighted model with adjustment of age, sex, BMI range, and SOFA score.

In a subgroup analysis with the univariate models, the association between FI and 28-day mortality was observed among patients with a normal BMI (18.5–25) (OR 1.577, 95% CI 1.111–2.238, *p* = 0.011), those primary diagnosis of respiratory disease (OR 1.761, 95% CI 1.136–2.728, *p* = 0.011), surgical patients (OR 2.024, 95% CI 1.001–4.096, *p* = 0.05), or patients with a SOFA score ≤ 8 (OR 1.701, 95% CI 1.118–2.588, *p* = 0.013) (Supplementary Figure S2).

**Figure 5:**
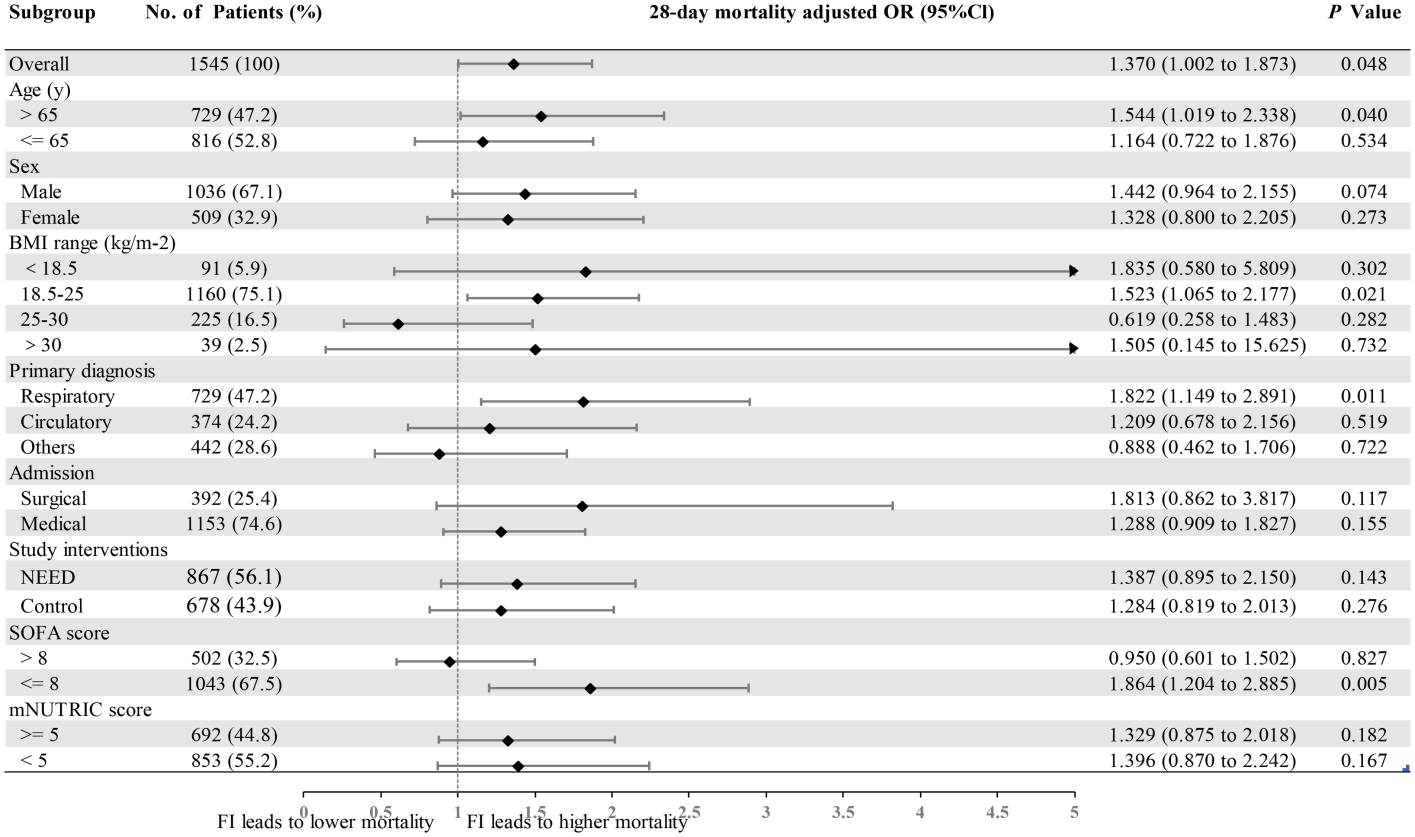
Feeding intolerance effects in different subgroups. Forest plot depicting the adjusted odds ratios (ORs) from a multilevel, multivariable analysis with a random effect on the centre of inclusion and adjustment of age, sex, body mass index (BMI) range and admission sequential organ failure assessment (SOFA) score. The association between FI and mortality at day 28 is assessed in subgroups according to sex, age, BMI range, primary diagnosis, surgical/medical patients, NEED/control group, SOFA score, and modified nutrition risk in critically ill (mNUTRIC) score.

In a subgroup analysis with the multivariable models, the association between FI and 28-day mortality was also observed in patients with a normal BMI (aOR 1.523, 95% CI 1.065–2.177, *p* = 0.021), patients with a primary diagnosis of respiratory disease (aOR 1.822, 95% CI 1.149–2.891, *p* = 0.011), or patients with a SOFA score ≤ 8 (aOR 1.864, 95% CI 1.204–2.885, *p* = 0.005). Additionally, a relationship between FI and 28-day mortality was also observed in the subgroup of patients aged over 65 years (aOR 1.544, 95% CI 1.019–2.338, *p* = 0.04). However, in the surgical patient subgroup, the association between FI and 28-day mortality was no longer significant (aOR 1.813, 95% CI 0.862–3.817, *p* = 0.117) (Figure 5). After BH-FDR adjustment for multiple subgroup comparisons, none of the subgroup-level associations remained statistically significant (all *q* values >0.05; Supplementary Table S2).

In addition, we also considered the effect of time variables on the outcome, we employed Kaplan–Meier survival curves and the Log-rank test to document the survival time up to 28 days after ICU admission. Ultimately, we plotted the Kaplan–Meier survival curves for the total patient cohort (Supplementary Figure S3) as well as for each subgroup (Supplementary Figure S4).

### 3.6 Association between FI and other short-term clinical outcomes

In other short-term clinical outcomes, patients with FI had longer ICU length of stay (LOS) (OR 1.903, 95% CI 0.436–3.307, *p* = 0.011) and shorter ICU-free days D28 (OR −0.919, 95% CI −1.763 to −0.076, *p* = 0.033) and Ventilator-free days D28 (OR −1.149, 95% CI −2.213 to −0.084, *p* = 0.035) compared to patients with non-FI. No association was found between the occurrence of FI and Albumin D10 (OR 0.119, 95% CI −0.654 to 0.893, *p* = 0.762) or Pre-albumin D10 (OR 0.007, 95% CI −0.007 to 0.022, *p* = 0.320) (Table 3).

## 4 Discussion

In this *post hoc* analysis of the NEED trial, we observed that in critically ill patients undergoing EEN, the occurrence of FI was associated with increased 28-day mortality, prolonged ICU LOS, and a reduction in both ICU-free days and ventilator-free days at 28 days. Notably, in the subgroup of patients aged over 65 years, with a normal BMI, a primary diagnosis of respiratory disease, and a SOFA score of ≤ 8, the association between FI and 28-day mortality was observed. However, after Benjamini–Hochberg FDR correction for multiple subgroup comparisons, these subgroup findings were no longer statistically significant and should therefore be interpreted with caution. In addition, no significant association was found between FI and levels of albumin and pre-albumin at day 10.

We focused this study on patients who initiated EEN in the NEED trial, as this group is of particular concern regarding the incidence of FI in clinical practice. While selecting total PN or delaying EN could potentially prevent FI, such approaches would contradict established guidelines and deviate from clinical practice; therefore, we excluded these patients from our analysis. As anticipated, the group experiencing FI had a lower average daily intake of energy and protein.

GI symptoms may occur during the delivery of EN, accompanied by GI dysfunction, and this dysfunction may develop or worsen due to EN. Focusing on the occurrence of FI may help reduce GI deterioration and improve prognosis. In this study, we reported the detailed process of FI evaluation and included all known data related to FI to increase the transparency of this study.

In addition, we also considered the potential influence of the feeding protocol intervention implemented in the NEED trial (11). We found that among the patients diagnosed with FI, the proportion of patients diagnosed with FI who met both the feeding dose and GI symptoms was relatively high, which is not unexpected, because most of the patients were due to GI symptoms leading to reduced feeding, resulting in substandard feeding doses. We also found that nutrient supply was similar between the NEED group and the control group, as was the distribution of AGI grades, and these results were consistent with those reported in the original study. Interestingly, there were some differences in the causes of FI diagnosis between the two groups: In the control group, the proportion of FI diagnosis was higher due to substandard feeding dose alone; In the NEED group, the proportion of FI diagnosed solely due to GI symptoms was higher, and the proportion of feeding dose and GI symptoms meeting the diagnostic criteria of FI was higher. These results may be related to more aggressive feeding strategies in the NEED group. The impact of these factors is likely to be small in this study because we are looking at which patients are more susceptible to the effects of FI.

The primary objective of this study was to explore the association between FI and short-term clinical outcomes. Although univariate analysis did not reveal an association between FI and 28-day mortality, multivariable logistic regression identified a significant association between FI and 28-day mortality, which remained robust in the propensity-weighted analysis. Reintam Blaser et al. (6) (*N* = 1712) found that FI was the strongest predictor of mortality within 90 days of hospitalization. Similarly, Heyland et al. (5) (*N* = 15,918) reported that among critically ill patients on mechanical ventilation, FI was associated with fewer ventilator-free days, longer ICU lengths of stay, and higher mortality. Our findings are consistent with these studies, suggesting that FI may lead to a deteriorated overall clinical status, potentially resulting in inadequate energy and protein intake and subsequent malnutrition, thereby impacting recovery from critical illness. As a result, patients experiencing FI tend to be more reliant on mechanical ventilation, have prolonged ICU LOS, and exhibit higher short-term mortality.

However, the study by Virani et al. found an association between FI and decreased serum prealbumin levels in trauma patients, a relationship that was not observed in our research (15). Prealbumin levels reflect a patient’s nutritional status to some extent; during the early phase of critical illness, infections can inhibit prealbumin synthesis, making this marker indicative of the severity of acute inflammatory responses (16). In our study, we monitored prealbumin levels on day 10, and lower prealbumin levels during the post-acute phase suggest reduced protein reserves and difficulties in recovery from illness. Although we identified poorer short-term outcomes in the FI group, we did not find a correlation between FI and prealbumin levels on day 10.

The second objective of this study was to investigate the association between FI and short-term mortality in specific populations of critically ill patients. The results aligned with our expectations: not all types of critically ill patients exhibited an association between FI and increased short-term mortality. In patients with a normal BMI, the occurrence of FI is more strongly associated with an increased 28-day mortality. GI failure caused by FI may be a major factor for poorer prognosis. Obese patients may be more tolerant of FI (one possible explanation is). Another possible explanation is that obese patients have more nutritional reserves, making them more tolerant of malnutrition caused by FI. In underweight patients, this association was not observed, which may be related to the smaller sample size; however, we believe that such an association could exist, for reasons similar to those seen in patients with a normal BMI.

Patients whose primary diagnosis is respiratory disease exhibited a lower incidence of FI. However, our subgroup analysis revealed that in this population, the occurrence of FI was more strongly associated with increased 28-day mortality. In the ICU, the impact of respiratory diseases on GI function may be relatively minimal, as hypoxic conditions may often be resolved quickly (17). With the improvement in blood oxygen saturation, GI dysfunction may recover within a short time frame. Nevertheless, when these patients do experience FI, it may indicate poorer GI function or even multi-organ dysfunction, thus providing an explanation for the stronger association between FI and 28-day mortality.

Interestingly, our subgroup analysis also found that in patients with a SOFA score ≤8, the association between FI and 28-day mortality was even more pronounced, which was somewhat unexpected. Typically, patients with milder conditions have a lower likelihood of experiencing FI. This aligns with the previous theory; however, when these patients do develop FI, it may suggest underlying GI dysfunction (an important prognostic factor not reflected in the SOFA score (18)) and a worse overall clinical status. In patients with higher SOFA scores, 28-day mortality is largely driven by severe multi-organ dysfunction, so the additional prognostic contribution of FI may be modest. These factors may help clarify this unexpected finding.

Although our univariate analysis did not reveal an association between FI and 28-day mortality in elderly patients, the multivariate analysis identified such an association. This may be because confounding factors may mask the true relationship between FI and mortality in univariate analysis. The greater impact of FI on elderly patients may be attributable to several factors, including higher prevalence of comorbidities, reduced recovery capacity, and impaired immune and metabolic functions, which collectively contribute to a lower tolerance for the adverse effects of FI. We will interpret this finding with caution. In addition, the subgroup classification and the selection of critical values in this study were not only based on the NEED dataset, but also referred to the FRANS study (19).

This study presents certain innovative aspects. We have validated previous findings that FI is associated with poorer short-term outcomes. Furthermore, we have identified a group of patients who are more sensitive to FI, indicating that the occurrence of FI may lead to worse prognoses in this population.

However, this study has some limitations. First, although it represents a secondary analysis of a cluster randomized controlled trial conducted across 97 ICUs in China, the study began in 2017, and current nutritional practices (such as progressive feeding) differ from those at that time. Consequently, some patients in our study may have experienced FI due to early excessive feeding; adherence to the feeding strategies recommended by current guidelines could potentially reduce the overall incidence of FI. We acknowledge that this limitation is significant. Second, we excluded patients who were receiving oral nutrition (albeit a small number), as their nutritional intake could not be accurately quantified. The study focused solely on nutritional calorie and protein intake, and due to a lack of data, we could not assess non-nutritive calories and proteins, such as those from propofol, which may lead to an underestimation of overall nutritional intake.

## 5 Conclusion

This study indicates that in critically ill patients initiated on EEN, the occurrence of FI was associated with higher 28-day mortality, longer ICU LOS, fewer ventilator-free days and ICU-free days. Elderly patients, those with a normal BMI, those with a primary diagnosis of respiratory diseases, and those with a SOFA score of ≤ 8 may be more susceptible to adverse effects of FI. However, these subgroup findings should be interpreted cautiously given multiple comparisons.

## Data Availability

The raw data supporting the conclusions of this article will be made available by the authors, without undue reservation.
