# Supplementary material for: Which critically ill patients are more susceptible to the adverse effects associated with feeding intolerance? A secondary analysis of a cluster-randomized controlled trial
Source: Front Nutr. 2026 Mar 23;13:1729192. doi: 10.3389/fnut.2026.1729192 (PMC13050722; doi:10.3389/fnut.2026.1729192)
Supplement: Supplementary file 1 [file Table_1.DOCX]

# Additional file 1

**Table S1.** **Multivariable logistic analysis of factors associated with feeding intolerance.** N=1545. Model adjusted for age, sex, BMI range, and admission SOFA score*.*

| Variable | aOR (95%CI) | *P* value |
| --- | --- | --- |
| Age (years) | 1.002 (0.995 - 1.009) | 0.555 |
| Sex |  |  |
| Male | Ref |  |
| Female | 0.631 (0.506 - 0.786) | < 0.001 |
| BMI range |  |  |
| < 18 | Ref |  |
| 18–25 | 3.717 (2.297 –6.222) | < 0.001 |
| 25–30 | 4.765 (2.796 - 8.359) | < 0.001 |
| > 30 | 10.251 (4.377 - 25.707) | < 0.001 |
| Admission SOFA score | 1.044 (1.011 - 1.077) | 0.008 |

Abbreviation: aOR: adjusted odds ratio, BMI: body mass index, SOFA: Sequential Organ Failure Assessment.

**Table S2. Multiple-comparison adjustment for subgroup analyses (BH-FDR)**

| Subgroup level | BH-FDR q value |
| --- | --- |
| Age >65 | 0.190 |
| Age ≤65 | 0.634 |
| Sex Male | 0.281 |
| Sex Female | 0.410 |
| BMI <18.5 | 0.410 |
| BMI 18.5–25 | 0.133 |
| BMI 25–30 | 0.410 |
| BMI >30 | 0.773 |
| Primary diagnosis: Respiratory | 0.104 |
| Primary diagnosis: Circulatory | 0.634 |
| Primary diagnosis: Others | 0.773 |
| Admission: Surgical | 0.346 |
| Admission: Medical | 0.346 |
| Study intervention: NEED | 0.346 |
| Study intervention: Control | 0.410 |
| SOFA >8 | 0.827 |
| SOFA ≤8 | 0.095 |
| mNUTRIC >5 | 0.346 |
| mNUTRIC ≤5 | 0.346 |

Notes: To address multiplicity across subgroup comparisons, Benjamini–Hochberg false discovery rate (BH-FDR) adjusted q values were calculated across all subgroup-level tests (n=19).
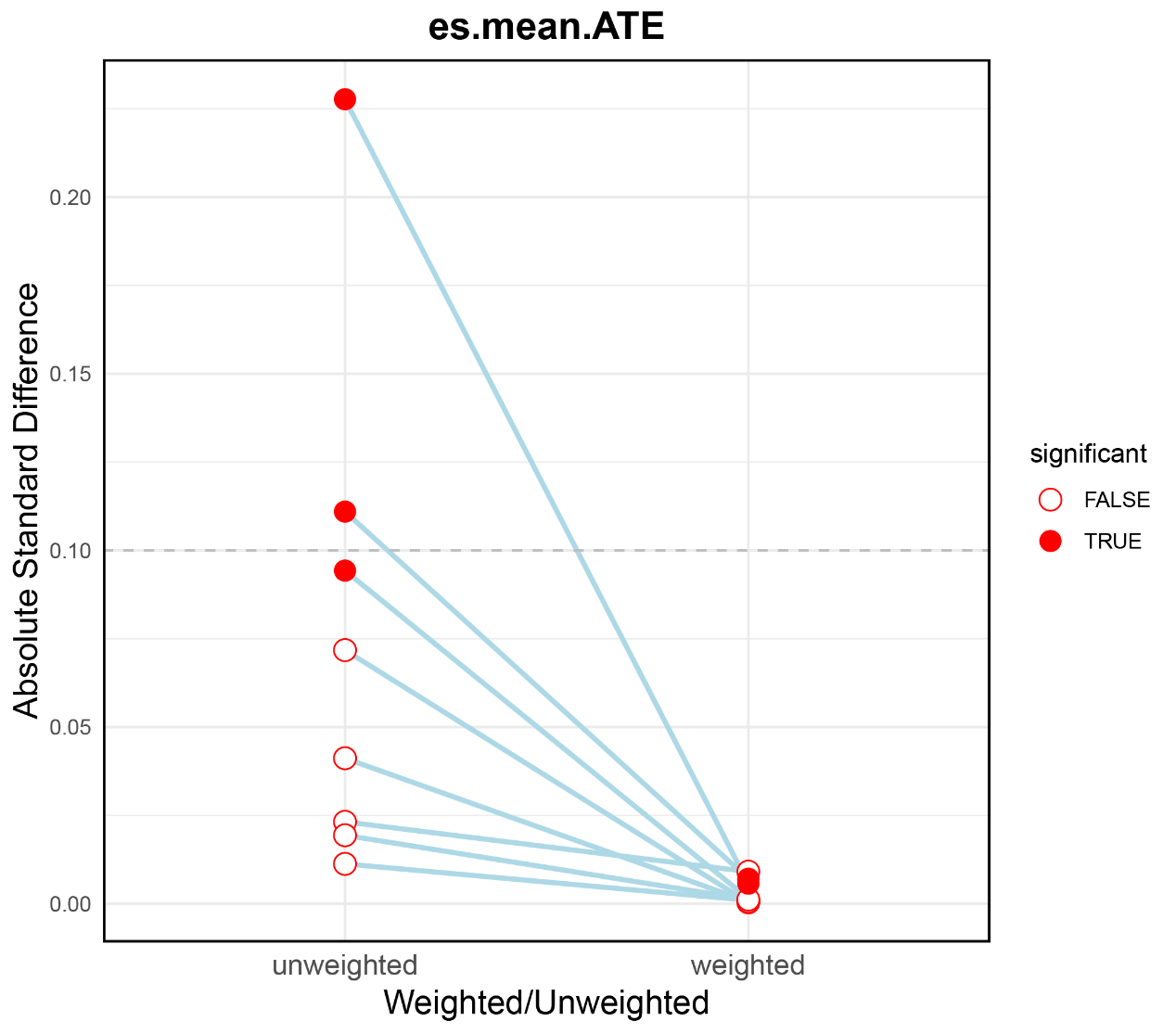


**Figure S1. Propensity score balance of feeding intolerance.** Comparisons of the absolute standardised mean differences (ASMDs) between the groups FI (Feeding intolerance) or non-FI on selected covariates (age, sex, BMI range, admission SOFA score), before and after weighting. After propensity score weighting, the ASMD decreases for all chosen covariates. No significant difference persists after weighting. Standardised effects of less than 0.20 are considered low (better balance), 0.40 as moderate and 0.60 as large.


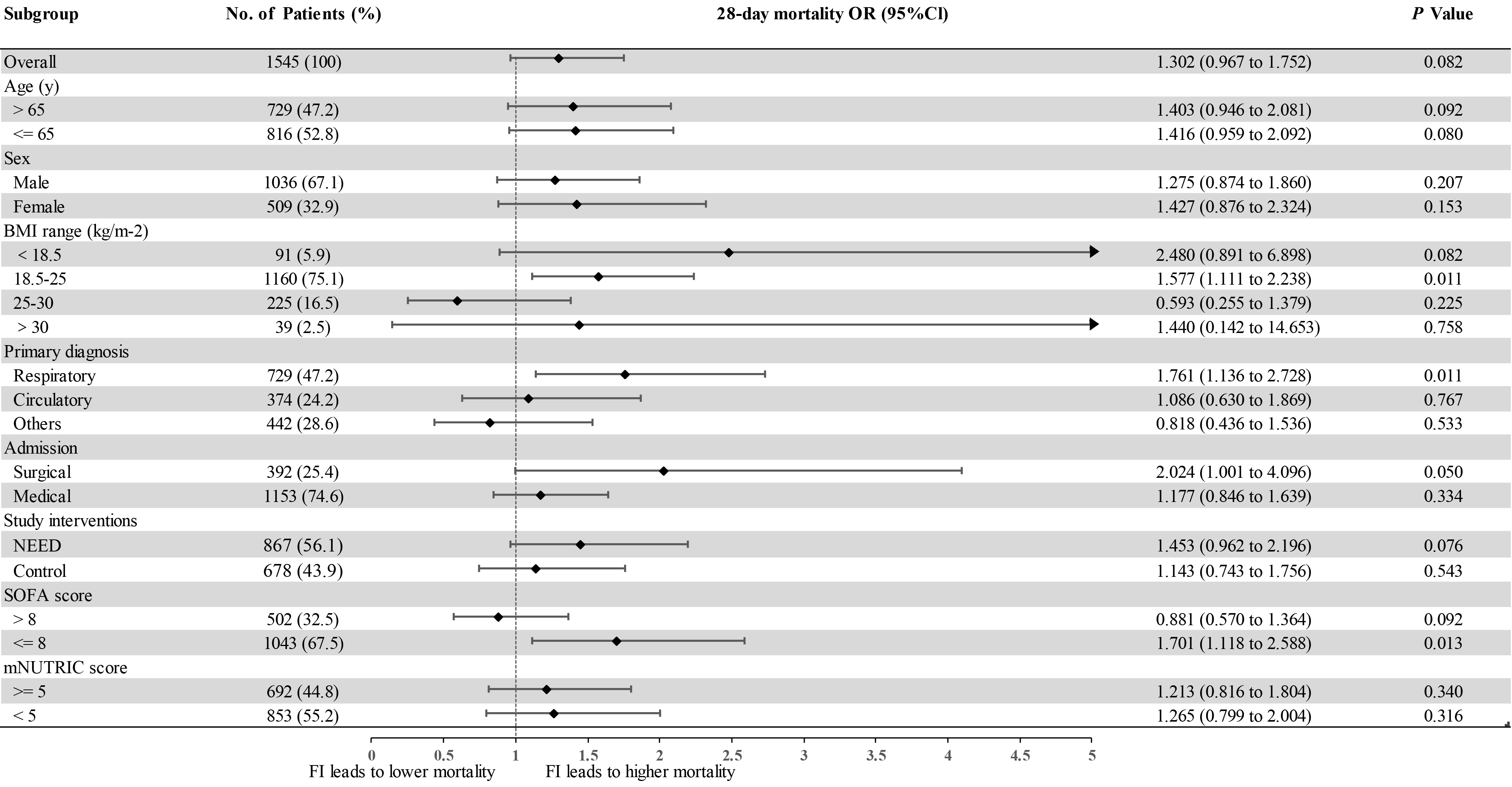


**Figure S2. Feeding intolerance effects in different subgroups.** Forest plot depicting the odds ratios (OR) from a univariate analysis. The association between FI and mortality at day 28 is assessed in subgroups according to sex, age, BMI range, Primary diagnosis, Surgical/medical patients, NEED/ control group, SOFA score and mNUTRIC score.


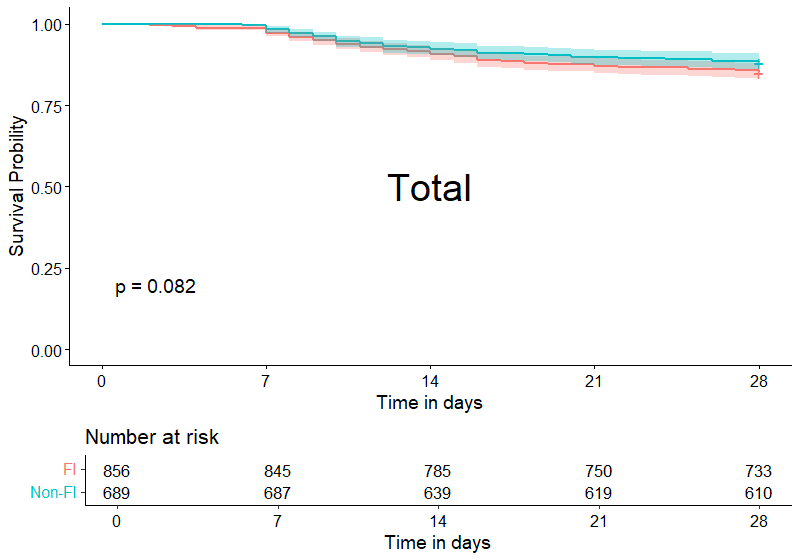


**Figure S3.** **28-day landmark-survival curves in the total study population.** Outcome was available for all patients. P-value determined by log-rank test. FI: Feeding intolerance.


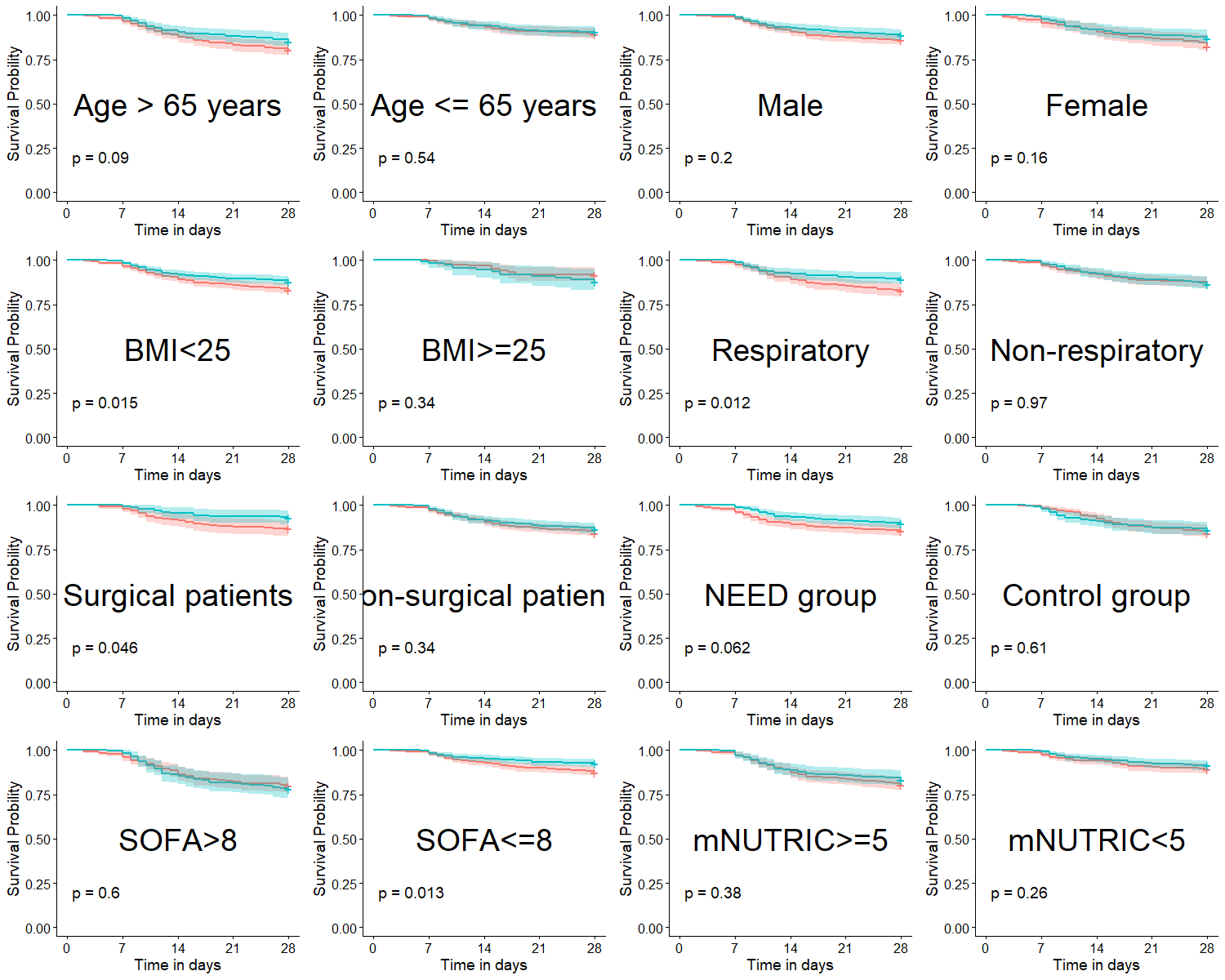


**Figure S4. 28-day landmark-survival curves in different subgroups.** Outcome was available for all patients. P-value determined by log-rank test. FI: Feeding intolerance; BMI: body mass index, SOFA: Sequential organ failure assessment; mNUTRIC: modified nutrition risk in critically ill.
